# Severe Dumping and Persistent Obesity in a Post-Roux-en-Y Gastric Bypass Patient With a Non-discernible Gastric Pouch and Symptomatic Improvement on Tirzepatide: A Case Report

**DOI:** 10.7759/cureus.113316

**Published:** 2026-07-24

**Authors:** Anthony L Frank, Idir Mohli, Navrooh Kaur, William Arnold

**Affiliations:** 1 Surgery, St. George's University School of Medicine, St. George's, GRD; 2 Surgery, Abrazo Arrowhead, Phoenix, USA; 3 Bariatric Surgery, Abrazo Arrowhead, Phoenix, USA

**Keywords:** dumping syndrome, revisional bariatric surgery, roux-en-y gastric bypass, tirzepatide, weight regain

## Abstract

Rapid transit of ingested nutrients into the small bowel following gastric or esophageal surgery can trigger dumping syndrome, a recognized postoperative complication seen with particular frequency after Roux-en-Y gastric bypass (RYGB). Under normal operative conditions, RYGB constructs a proximal gastric pouch that serves to limit meal volume and pace nutrient delivery into the jejunum.

This report describes a 65-year-old woman with a history of RYGB and subsequent surgical revisions who presented with debilitating postprandial symptoms consistent with dumping syndrome alongside persistent class III obesity. Upper endoscopy revealed no identifiable gastric pouch or gastric mucosa, with jejunal tissue encountered immediately distal to the esophagus. Her clinical course was notable for poor food tolerance, absent satiety, and substantial weight recidivism. After starting tirzepatide she demonstrated meaningful symptomatic relief alongside a BMI reduction from 46 to 41.9.

Interpretation of this case is limited by the unavailability of operative records and the absence of objective diagnostic workup, precluding any definitive conclusions about causation. Nevertheless, the case draws attention to an uncommon postoperative anatomical configuration that appears to contribute to both severe dumping physiology and failure of weight maintenance. Additionally, the clinical response observed with tirzepatide raises the possibility of a therapeutic application in this patient population, pending more robust evidence.

## Introduction

Dumping syndrome is a postprandial disorder that occurs after gastric or esophageal surgery due to rapid delivery of nutrients into the small intestine [1]. It is typically classified into early and late forms. Early dumping occurs within 30 minutes of eating and is mediated by osmotic fluid shifts and vasoactive peptide release, whereas late dumping is characterized by postprandial hyperinsulinemic hypoglycemia [1,2].

Roux-en-Y gastric bypass (RYGB) significantly alters gastrointestinal anatomy and physiology, predisposing patients to dumping syndrome [3,4]. The procedure involves the creation of a small gastric pouch that restricts intake and regulates nutrient flow into the jejunum [3].

Although dumping syndrome after RYGB is well described, the functional consequences of markedly altered anatomy following revisional bariatric surgery remain incompletely characterized [5]. Weight regain after RYGB is also a recognized long-term complication and may prompt further intervention [6].

We present a case of severe dumping-type symptoms in a patient with endoscopic findings consistent with a non-discernible gastric pouch following RYGB. Initiation of tirzepatide was associated with marked improvement of symptoms and BMI.

## Case presentation

A 65-year-old female with a history of morbid obesity underwent RYGB in 2007 with an approximate 200-pound weight loss. She later underwent multiple surgical interventions, including hiatal hernia repair and attempted revision of her bypass; however, detailed operative records were unavailable.

**Figure 1:**
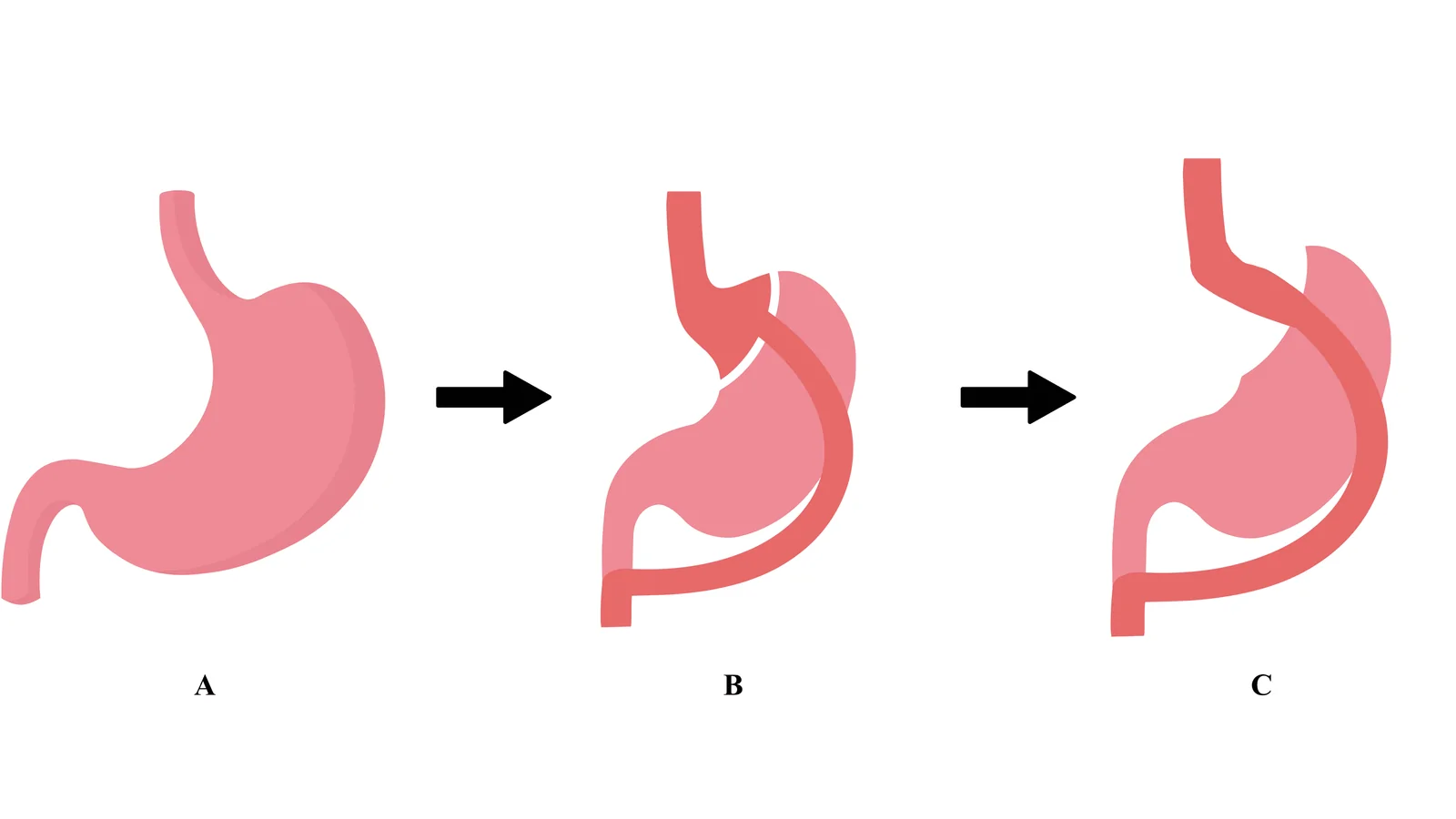
Schematic comparison of gastric anatomic configurations. (A) Normal stomach anatomy, with continuity between the esophagus, stomach, and duodenum. (B) Standard RYGB anatomy, showing a small proximal gastric pouch anastomosed to the Roux (alimentary) limb, with the excluded stomach and biliopancreatic limb diverted separately. (C) Suggested postoperative anatomy in this patient, based on endoscopic findings, illustrating an apparent direct transition from the esophagus into jejunal tissue without a discernible gastric pouch Figure created by the authors using Canva RYGB: Roux-en-Y gastric bypass

For anatomical orientation, Figure 1 provides a schematic comparison of normal gastric anatomy, standard RYGB configuration, and the proposed postoperative anatomy identified in this patient.

The patient developed persistent symptoms clinically consistent with dumping syndrome, including postprandial discomfort, intolerance to multiple foods, and impaired satiety; symptoms occurred in close temporal relation to meals, though formal classification into early versus late dumping was not established. No formal diagnostic testing such as provocative testing or continuous glucose monitoring was performed.

Despite having undergone prior bariatric surgery, she experienced significant weight regain, with a BMI of 46 recorded at the time of evaluation.

**Figure 2:**
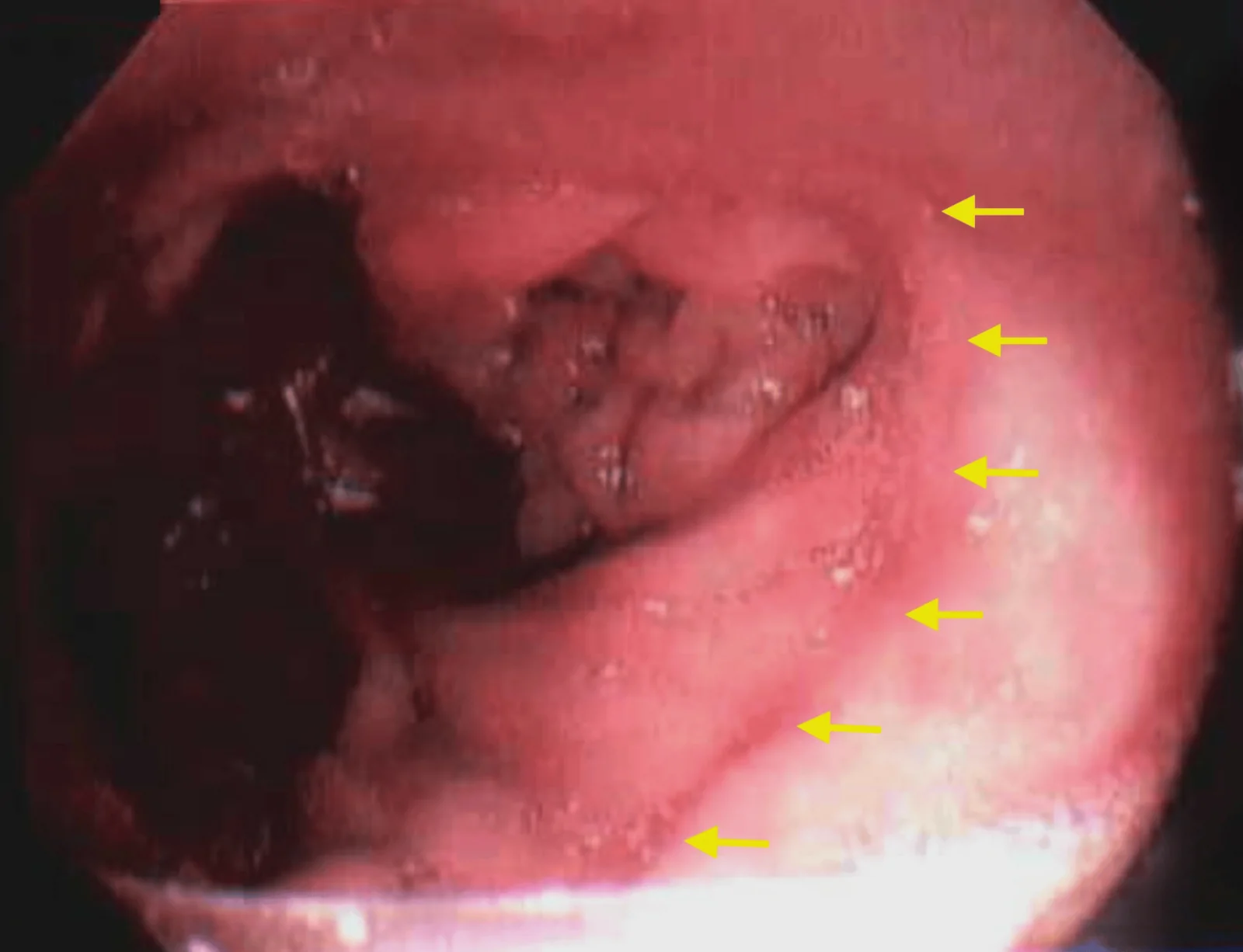
Endoscopic view demonstrating the transition zone between visualized mucosa, with arrows outlining the irregular margin along which no discernible gastric pouch or gastric mucosa could be identified.

**Figure 3:**
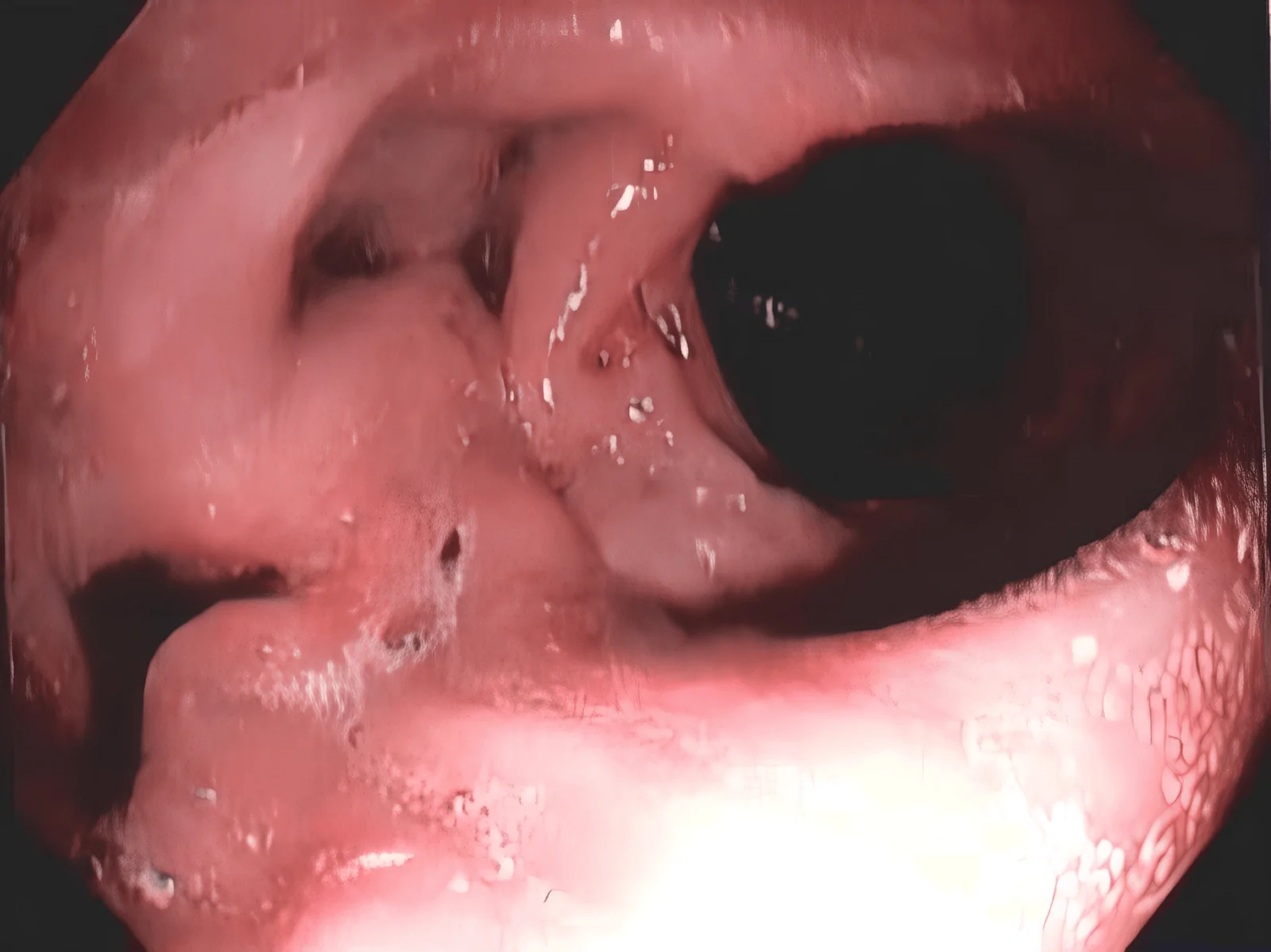
Endoscopic view showing apparent direct transition from esophagus to jejunal mucosa without a discernible gastric reservoir, consistent with altered postoperative anatomy.

Esophagogastrojejunoscopy performed in August 2022 demonstrated normal esophageal mucosa; upon advancement of the scope beyond the esophagus, jejunal mucosa was immediately encountered. No discernible gastric pouch or gastric mucosa was identified. There were no ulcerations, strictures, or inflammatory changes. These findings were interpreted as consistent with markedly altered postoperative anatomy. Representative endoscopic images are shown in Figures 2-3.

The anatomical configuration was interpreted as consistent with Roux-en-Y reconstruction but without a clearly identifiable gastric reservoir.

The patient was started on tirzepatide 2.5 mg subcutaneously once weekly in 2025, with follow-up scheduled every three months. At the most recent recorded follow-up, her BMI had improved from 46 to 41.9, and she reported marked improvement in postprandial symptoms and improved tolerance to oral intake. Subsequent dose titration and longer-term follow-up data were not available to the authors.

## Discussion

Dumping syndrome is a disorder of abnormally rapid nutrient delivery into the small intestine after gastric or esophageal surgery. In normal anatomy, the stomach acts as both a reservoir and a regulator: it stores food, triturates solids, mixes chyme, and releases contents into the duodenum in a controlled fashion. After operations that reduce gastric volume or bypass the pylorus, that buffering function is lost or diminished. Hyperosmolar food can then enter the small bowel too quickly, drawing fluid into the intestinal lumen and triggering release of vasoactive and incretin hormones. This produces the classic manifestations of early dumping - abdominal cramping, bloating, diarrhea, tachycardia, palpitations, flushing, dizziness, and fatigue, typically within 10-30 minutes of eating. Late dumping occurs later, usually 1-3 hours after a meal, and is driven primarily by an exaggerated insulin response after rapid glucose absorption, causing postprandial hypoglycemia [1,2].

This mechanism is highly relevant to the present case. Standard RYGB creates a small proximal gastric pouch that is connected to the Roux limb while the remaining stomach, duodenum, and proximal jejunum are excluded from nutrient flow [3]. Even though RYGB already predisposes to faster nutrient transit than native anatomy, the pouch still serves an important mechanical role: it limits meal volume and contributes to early satiety [3]. As previously noted, RYGB alters gastric emptying, intestinal exposure to nutrients, bile acid signaling, and gut hormone release, particularly GLP-1 and peptide YY [4].

What makes this case unusual is that the endoscopist documented immediate encounter of jejunal mucosa without an identifiable gastric pouch or visible gastric mucosa, suggesting a profoundly altered postoperative anatomy rather than a typical bariatric RYGB configuration. It is important to note that the available evidence supports endoscopic findings consistent with a non-discernible gastric pouch, rather than definitive proof of total pouch absence. That distinction matters because the original operative records and revision details are unavailable. Similar principles are seen in other post-gastrectomy or esophagojejunal reconstructions [7].

The coexistence of severe dumping-type symptoms and persistent morbid obesity represents a clinically important paradox. Rapid transit can worsen dumping while loss of a functional reservoir can simultaneously promote inadequate satiety and facilitate weight regain [6,8].

Post-bariatric physiology exists along a spectrum that includes dumping syndrome, altered incretin signaling, and postprandial hypoglycemia. Rapid delivery of nutrients to distal bowel segments results in increased secretion of incretin hormones such as GLP-1, contributing to exaggerated insulin responses [2,9].

Tirzepatide is a dual glucose-dependent insulinotropic polypeptide (GIP) and glucagon-like peptide-1 (GLP-1) receptor agonist that exerts its effects through coordinated actions on pancreatic, gastrointestinal, and central nervous system pathways [10-12]. At the pancreatic level, activation of GLP-1 receptors enhances glucose-dependent insulin secretion while suppressing glucagon release, thereby improving postprandial glycemic control without causing inappropriate hypoglycemia [12]. Concurrent activation of GIP receptors further augments insulin secretion in response to nutrient intake and may enhance β-cell responsiveness and insulin sensitivity [11,13].

At the gastrointestinal level, GLP-1 receptor activation plays a critical role in modulating gastric motility and nutrient transit. Specifically, GLP-1 slows gastric emptying through multiple mechanisms, including relaxation of the gastric fundus, inhibition of antral contractility, and increased pyloric tone, resulting in delayed delivery of nutrients into the small intestine [14,15]. Tirzepatide has been shown to transiently delay gastric emptying in a manner similar to GLP-1 receptor agonists, particularly early in therapy, which contributes significantly to reductions in postprandial glucose excursions [14,15].

At the central level, GLP-1 receptors located in the hypothalamus and brainstem regulate appetite and energy balance. Activation of these pathways leads to reduced caloric intake, increased satiety, and altered food reward signaling, contributing to sustained weight loss [10,16].

Taken together, these mechanisms provide a biologically plausible explanation for the observed improvement in dumping-type symptoms. First, by slowing gastric emptying and modulating gastrointestinal motility, tirzepatide may reduce the rate and magnitude of nutrient delivery to the small intestine, thereby attenuating the osmotic and neurohormonal cascade responsible for early dumping [14,15]. Second, by improving postprandial glucose handling and reducing glycemic variability, it may blunt exaggerated insulin responses and mitigate hypoglycemia-related symptoms [12]. Third, enhanced satiety and reduced caloric intake may partially compensate for the loss of restrictive anatomy [10].

Although these mechanisms are well supported physiologically, clinical evidence for the use of tirzepatide in dumping syndrome remains limited to case reports and small observational data [14,16]. Therefore, its role should be interpreted as a mechanistically supported but not yet guideline-established therapeutic option.

In this patient, tirzepatide was associated with marked improvement in symptoms and modest weight reduction. While this supports a potential therapeutic role, causality cannot be established.

The consensus management of dumping syndrome begins with dietary modification, followed by pharmacologic therapy such as acarbose or somatostatin analogues in refractory cases, and endoscopic or surgical interventions in selected patients [1,17]. Tirzepatide is not currently part of standard treatment algorithms and should therefore be viewed as an observed therapeutic benefit rather than an established therapy.

Revisional bariatric surgery may result in complex and unpredictable postoperative anatomy [5,18]. This case highlights the importance of reassessing gastrointestinal anatomy in patients with persistent or atypical symptoms following bariatric surgery.

This case has several limitations. Operative records were unavailable, limiting definitive characterization of the patient’s postoperative anatomy. There was no imaging confirmation such as contrast studies, and no objective diagnostic testing (e.g., mixed-meal testing or continuous glucose monitoring) to confirm dumping syndrome or distinguish between early and late forms. The observed response to tirzepatide is based on clinical observation without controlled comparison, and causality cannot be established. Additionally, the authors did not have access to follow-up data beyond the documented visit, so the durability of symptomatic and weight-related improvement, as well as any subsequent dose adjustments, could not be assessed.

## Conclusions

This case highlights a rare and clinically complex postoperative presentation in which the apparent absence of a functional gastric pouch was associated with both severe dumping-type symptoms and persistent morbid obesity following RYGB and multiple revisions. The simultaneous occurrence of these two conditions reflects the physiological paradox that can arise when revisional bariatric surgery results in markedly altered gastrointestinal anatomy.

These findings underscore the importance of thorough endoscopic anatomical reassessment in bariatric patients presenting with persistent or atypical postoperative symptoms, particularly those with complex revision histories and unavailable operative records, as standard symptom-based evaluations alone may be insufficient in this population.

The clinical response observed with tirzepatide suggests a potential therapeutic role in patients with severely altered post-bariatric anatomy, likely mediated through its effects on gastric transit, glycemic regulation, and satiety. However, causality cannot be established from a single case, and further prospective study is needed before tirzepatide can be considered an established treatment for dumping syndrome in this setting.
